# Aberrant expression of plasma microRNA-33a in an atherosclerosis-risk group

**DOI:** 10.1007/s11033-016-4082-z

**Published:** 2016-09-23

**Authors:** Soo Hwan Kim, Gi Jin Kim, Tsukuru Umemura, Seung Gwan Lee, Kyung Jin Cho

**Affiliations:** 1 Department of Integrated Biomedical and Life Science, Graduate School, Korea University, Hana Science Hall, Anam-Ro 145, Sungbuk-Gu, Seoul, 02841 South Korea; 2 Placenta Research Laboratory, Department of Biomedical Science, CHA University, 335 PanGyo-Ro, BunDang-Gu, SungNam-si, GyungGi-Do, 13488 South Korea; 3 Faculty of Medical Sciences, Department of Health Sciences, Kyushu University, Maidashi 3-1-1, Higashi-Ku, Fukuoka, 812-8582 Japan; 4 Faculty of Health and Environmental Science, College of Health Science, Korea University, Hana Science Hall, Anam-Ro 145, Sungbuk-Gu, Seoul, 02841 South Korea; 5 Department of Biomedical Laboratory Science, Gimcheon University, 214 Daehak-ro, Gimcheon City, Gyeongsangbuk-Do 39528 South Korea; 6 Department of Medical Technology and Sciences, International University of Health and Welfare, Enokizu 137-1, Ohkawa City, Fukuoka 831-8501 Japan

**Keywords:** Plasma biomarker, MiRNA-33a, ABCA1, MicroRNA oligonucleotides, Atherosclerosis, Foam cells

## Abstract

In order to investigate whether plasma microRNA-33a (miR-33a) can be a biomarker for the early detection of atherosclerosis and to reexamine the assumption that miR-33a represses the expression of ABCA1, we compared the expression levels of miR-33a and ATP-binding cassette A1 (ABCA1) using human plasma and supernatants of macrophage cultured media. We first separated ample number of plasma samples from left-over whole blood samples based on the criteria for normal or dyslipidemia, and stored them at −20 °C until use. Then we selected 18 plasma samples for each normal, athero-risk and treated group using a metabolic disease cohort in which candidate subjects have participated. For classifying into three groups, we primarily relied on the records of physicians’ comments, prescriptions, treatment history, lipid profiles and test results from medical equipment aimed at the diagnosis for atherosclerosis or cardiovascular disease. After collecting the final 54 plasma samples, we analyzed and compared the expression levels of miR-33a and ABCA1 at the plasma levels. In the comparison of plasma levels of the three groups, the miR-33a expression level of athero-risk group was 5.01-fold higher than that of normal group. Meanwhile, in the culture of foam cells transfected with anti-miR-33a oligonucleotides, the miR-33a level significantly decreased, while ABCA1 level significantly increased. The results suggest that enhanced expression of miR-33a might induce cholesterol accumulation and aggravate inflammation in vessel walls by suppressing the expression of ABCA1 in macrophages. Thus, plasma miR-33a can be considered as a candidate biomarker of atherosclerosis.

## Introduction

In the development of atherosclerotic lesions, macrophages accumulate in the intima of the artery. Early lesions are rarely detected in atherosclerosis as the apoptotic cells are cleared by efferocytosis. When macrophages participate in the formation of atherosclerotic plaques, they appear to have a decreased capacity to migrate, which contributes to failure to resolve inflammation and makes smooth muscle cells participate in the inflammatory process. In these advanced plaques, macrophages secrete inflammatory cytokines and bring about necrosis and apoptosis [1, 2], and the fibrous cap becomes thinner and prone to rupture, thereby causing thrombosis [3].

During reverse cholesterol transport (RCT), ATP-binding cassette A1 (ABCA1) moves cholesterol and phospholipids from the inner to the outer leaflet of the membrane, from where apolipoprotein A-1 (ApoA-1) carries the lipid components to nascent high-density lipoprotein (HDL) to generate larger HDL [4, 5]. In transgenic mice, the over-expression of human ABCA1 resulted in increased cholesterol efflux from macrophages. This implies that enhanced ABCA1 activity can protect against atherosclerosis in vivo [6].

**Fig. 1:**
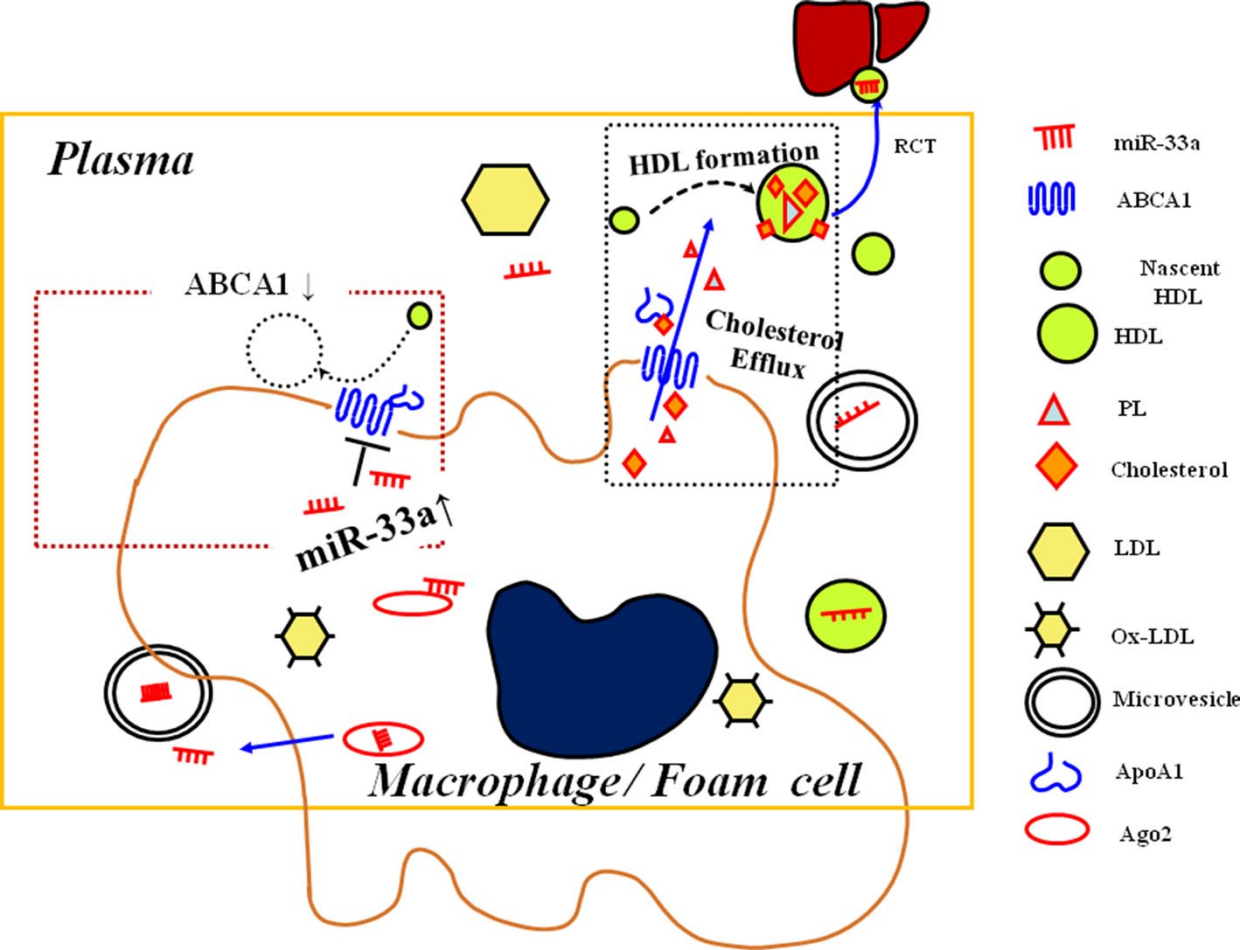
Elevated miR-33a represses the expression of ABCA1, leading to decreased HDL formation. Under normal circumstances, ABCA1 moves cholesterol and phospholipids from the *inner* to the *outer* leaflet of the macrophage membrane, thereby facilitating HDL formation. During the progression of atherosclerosis, aberrantly elevated miR-33a represses ABCA1 expression. Under conditions of decreased expression of ABCA1, the cholesterol efflux cannot function properly, resulting in decreased HDL formation. miR-33a might be secreted into plasma or transported to the liver via microvesicles, Ago2 or HDL

If expression of microRNA-33a (miR-33a) is aberrantly increased in macrophages, ABCA1 expression would be downregulated and the RCT may not function properly. In hepatocytes and macrophages, ABCA1 is antithetically expressed to the expression of miR-33a (Fig. 1) [7, 9, 10, 12, 17].

The over-expression of miR-33a/b reduces both fatty acid oxidation and insulin signaling in hepatic cell lines [7–10]. However, when an anti-miR-33a oligonucleotide was delivered into cells, the inhibitory effect of miR-33a on ABCA1 expression was alleviated [11, 12]. Thus, anti-miR-33a oligonucleotides are being investigated as a therapeutic tool to enhance either ABCA1 or HDL expression [13–18].

miRNAs maintain their stability under harsh conditions such as high temperature, extreme-pH values, storage at room temperature for extended periods, and repeated freeze–thawing cycles. Moreover, they are stable even in RNase-rich plasma, probably because they are sequestered within exosomes, microvesicles, or associated with either Ago2 or HDL [19–21]. Compared to large molecular weight plasma RNA, the exosomal miRNAs and plasma miRNAs were stable under different storage conditions and there were no significant influences on plasma miRNAs [22]. Serum miRNAs were also resistant to repeated freeze–thaw cycles. When serum was treated for 3 h in low (pH = 1) or high (pH = 13) pH solutions, miRNAs remained stable [23, 24]. Therefore, plasma, like serum, is an excellent source of miRNAs for research on hyperlipidemia and coronary artery disease [25]. Accordingly, this study investigated whether plasma miR-33a can be used as a diagnostic marker for the early detection of atherosclerosis.

## Materials and methods

### Sample selection criteria and classification of groups

Among the individuals who visited a laboratory center for medical checkups between February 21 and March 31, 2013, we carefully selected final 54 subjects who were participating in a metabolic disease cohort and had signed informed consent to allow us to use their left-over blood specimens. We obtained informed consent from medical check-up examinees at the beginning of their participation in the metabolic disease cohort and we have complied with the ethical principles outlined in the Declaration of Helsinki.

#### Primary sample selection and separation of plasma samples

First, at chemistry section on-the-spot, the subjects were classified into two groups ‘Normal’ and ‘Hyperlipidemic’, based on the criteria of five lipid parameters such as, total-cholesterol (T-cholesterol), low-density lipoprotein (LDL), HDL, triglycerides (TG), and the T-cholesterol/HDL ratio. If a sample subject met four of the five requirement criteria, then the subject was classified into ‘Normal’ group, and if met three of the hyperlipidemic criteria, then classified into ‘Hyperlipidemic’ groups (Table 1).

**Table 1:** Sampling criteria of normal, atherosclerosis-risk, and treated groups

Groups	1° selection criteria(based on on-the-spot test results of 5 lipid parameters)	2° selection criteria (based on records of physician comments, test results and cohort data)
Normal	T-Chol <200 mg/dL	*Normal group* No significant physicians’ clinical opinions along with at least four of the five lipid items should be within reference ranges of the laboratory (left)
	LDLc <130 mg/dL	
	HDLc ≥40 mg/dL (m)	
	≥50 mg/dL (f)	
	TG <150 mg/dL	
	TC/HDL ratio <3.5	
Hyperlipidmic	T-Chol ≥240 mg/dL	*Athero-risk group* Physicians’ temporary decisions on the cardiovascular status with respect to atherosclerosis (CVD, IHD, MI). Never treated or prescribed for the past five years on the above disease AND at least three of the five lipid items lie out of the laboratory reference ranges (left) *Treated group* Any treatment records and prescription history related with above diseases during for the past five years
	LDLc ≥160 mg/dL	
	HDLc <40 mg/dL (m)	
	<50 mg/dL (f)	
	TG ≥200 mg/dL	
	TC/HDL ratio ≥5.0	

*CVD* cardiovascular disease, *IHD* ischemic heart disease, *MI* myocardial infarction, *T-chol* total cholesterol, *LDLc* low-density lipoprotein cholesterol, *HDLc* high-density lipoprotein cholesterol, *TG* triglycerides

Following ‘on-the-spot’ dyslipidemia classification, two technologists at hematology section selected every left-over whole blood samples of the corresponding individual, and separated plasma fraction from the whole blood samples by centrifugation at 800×*g* for 20 min within 3 h after blood collection. Then, selected candidate plasma samples were stored at −20 °C one by one.

#### Final collection of 54 plasma samples

**Fig. 2:**
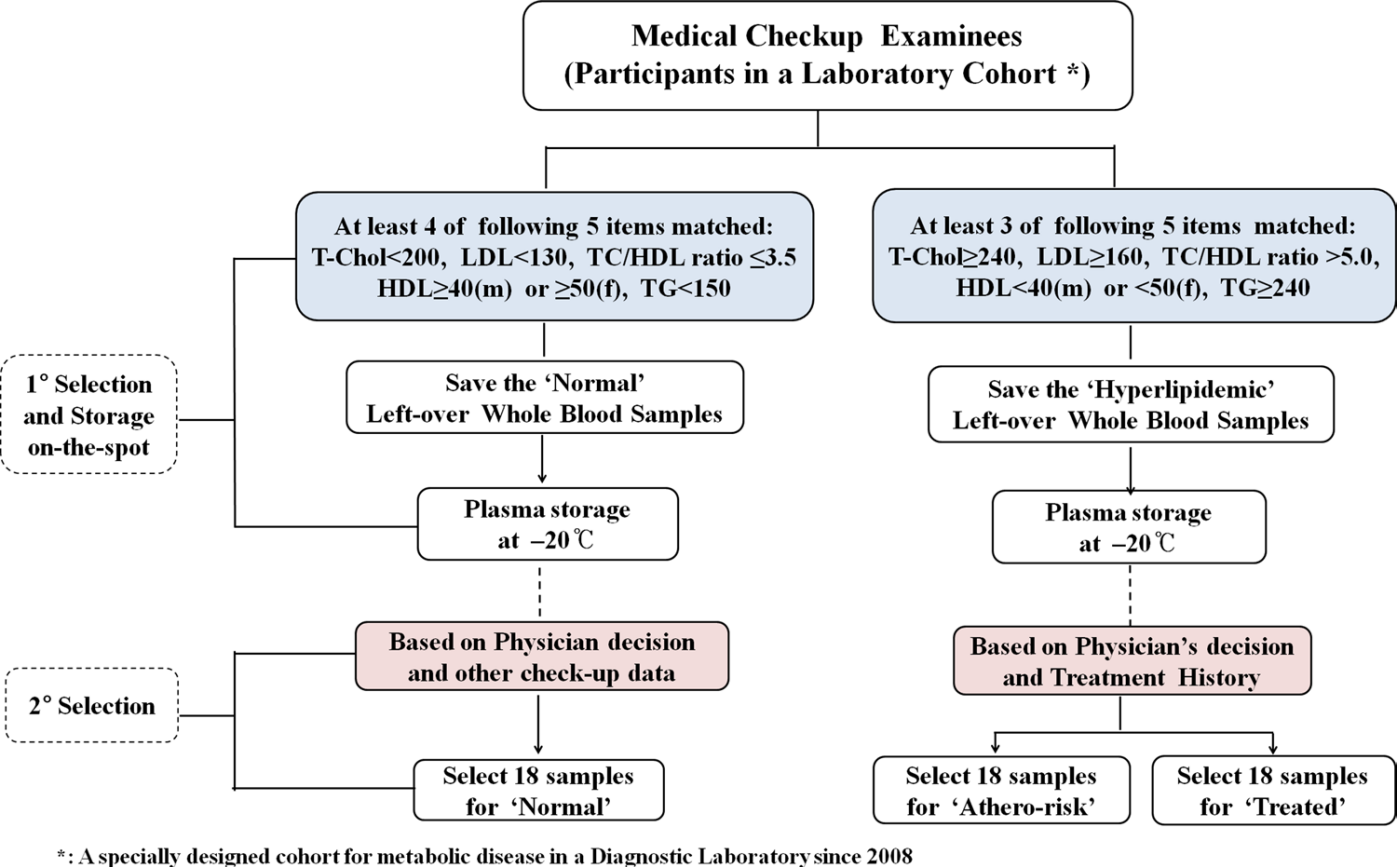
Two-step selection processes for 54 samples. First, in order to sort out normal and hyperlipidemic samples, the dyslipidemic individuals were selected according to the criteria of serum lipid levels. The left-over whole blood samples of target individuals were carefully selected, and separated plasma samples were stored in a deep freezer until use. In the secondary selection, the hyperlipidemic samples were further classified into ‘treated’ or ‘non-treated athero-risk’ ones based on the records of physicians’ comments on the individual’s status with respect to atherosclerosis, cardiovascular disease, ischemic heart disease, and myocardial infarction

For the classification of the subjects into Normal, Treated and Atherosclerosis-risk (Athero-risk) groups, we primarily focused on the records of physicians’ comments on the individual health status with respect to atherosclerosis, cardiovascular disease, ischemic heart disease and myocardial infarction. For better selection and discrimination of the groups, we attentively reviewed individuals’ cohort records on treatment history, prescriptions, and various tests such as, thermal conductivity, carotid ultrasound, pulse wave velocity, abnormal Q-wave, high levels of LDL, TG, fasting blood sugar (FBS), creatine kinase, and T-cholesterol/HDL ratio in addition to the physicians’ opinion. For a study on miR-33a as a candidate biomarker of atherosclerosis, we finally collected 18 samples for each group. The collected plasma samples were kept at −20 °C until use (Fig. 2).

### Isolation of miRNAs from plasma samples and culture media

miRNAs were isolated from human plasma samples or supernatant of foam cell culture. The frozen plasma and supernatants were thawed at 42 °C for 1 h. At the beginning of the study, the stabilities of miRNAs were verified using a batch of miRNAs such as miR-16, miR-33a, and miR-33b under the above conditions. Then, 400 μL of plasma or supernatant was treated with 600 μL Isogen (NipponGene, Toyama, Japan) at room temperature for 5 min, followed by 200 μL chloroform (Sigma–Aldrich, St Louis, MO, USA) and incubated for 10 min at room temperature. The sample was centrifuged at 15,000×*g* for 15 min, the upper aqueous phase was separated, and 500 μL isopropyl alcohol (Sigma–Aldrich) was added before incubating at room temperature for 15 min. After incubation, the sample was centrifuged again to precipitate RNA. The pellet was dried completely and dissolved in diethylpyrocarbonate-treated water. RNA concentrations were measured using a NanoDrop (Thermo Fisher Scientific, San Jose, CA, USA) instrument.

### Quantification of miR-33a levels in human plasma and supernatant of foam cell cultures

Total RNA from samples were reverse transcribed using Mir-X™ miRNA First-Strand Synthesis Kit (Clontech, Mountain View, CA, USA) and real-time PCR was performed using SYBR® Advantage® qPCR Premix (Clontech). PCR primers for miRNA-33a were prepared as follows: miRNA-33a forward, 5′-GTGCATTGTAGTTGCATTGCA-3′ [11, 12, 15], while the 3′ primer was included in the kit as a reverse primer. PCR was performed using a Rotor-Gene 6000 (Corbett Life Science, Concorde, NSW, Australia). As an internal control, a known amount of synthetic U6 snRNA was added to each sample, which was evaluated using Clontech Mir-S miRNA First Strand Synthesis and a SYBR RT-PCR kit. We calculated the ∆Ct of miR-33a in each sample using the internal control, U6 snRNA, by subtracting the Ct of U6 from the Ct of miR-33a. Then, we calculated the respective ∆∆Ct in each sample by subtracting the mean ∆Ct of the normal group from the ∆Ct of each sample. We calculated the 2^−∆∆Ct^ values for each sample and determined the mean value for each group. Finally, we compared the expression levels of miR-33a in plasma of three groups and in culture supernatant of five types of macrophages.

### Quantification of ABCA1 levels in human plasma and supernatant of foam cell cultures

ABCA1 concentration was measured using an ABCA1 ELISA kit (Cusabio, Wuhan, China) as per the manufacturer’s instructions. Briefly, human plasma samples were diluted with sample diluents (1:200) and 100 μL of each sample and the standard were added into the wells of a 96-well plate. The plate was incubated at 37 °C for 2 h and 100 μL of biotin-antibody was added to each well. The plate was incubated at 37 °C for 1 h, and each well was washed with wash buffer. One hundred microliters of horseradish peroxidase-avidin was loaded to each well and incubated at 37 °C for 1 h. For supernatant of foam cell culture, each supernatant sample was aspirated and washed five times with wash buffer, 90 µL tetramethylbenzidine substrate was added, and the samples were incubated at 37 °C for 20 min. After incubation, 50 μL stop solution was added immediately. ABCA1 concentrations were calculated from the optical density value of the sample and a standard curve.

### THP-1 cell culture, differentiation into macrophages, and preparation of ox-LDL

THP-1 cells were cultured in RPMI1640 medium (Hyclone, Logan, UT, USA) supplemented with 10 % (v/v) fetal calf serum (Gibco, Uxbridge, UK) at 37 °C in an atmosphere containing 5 % CO_2_. The THP-1 cells were induced to differentiate into macrophages by the addition of 250 ng/mL phorbol 12-myristate 13-acetate (PMA, Sigma–Aldrich, St Louis, MO, USA) for 72 h. Human LDL (Sigma–Aldrich) was oxidized with 7.5 μmol/L CuSO_4_ (Sigma–Aldrich) at 37 °C for 24 h. The oxidative reaction was stopped by the addition of 0.01 mmol/L EDTA. The lipoprotein concentration in each sample was determined by a BCA protein assay kit (Thermo Fisher Scientific), and oxidized-LDL was stored at 4 °C before use.

### Transfection with miRNAs and foam cell induction

To reexamine the assumption that miR-33a suppresses the expression of ABCA1 in macrophages, we first designed five types of macrophages (untreated macrophages; control foam cells treated with ox-LDL but not-transfected; and three batches of foam cells transfected with different miRNA oligonucleotides as follows: the first group of foam cells were transfected with 10 nM ‘miR-33a-mimic’ (5′-GTGCATTGTAGTTGCATTGCA-3′), the second group transfected with ‘anti-miR-33a’ (5′-TGCAATGCAACTACAATGCAC-3′), and the third group transfected with ‘miR-33a-mismatched’ (5′-TCCAATCCAACTTCAATCATC-3′) (Bioneer, Daejeon, Korea). The miRNA transfections were carried out with Lipofectamine RNAiMAX reagent for 24 h (Invitrogen, Carlsbad, CA, USA) in OptiMEM medium (Invitrogen). Then, the foam cells were prepared by treating the macrophages with 50 μg/mL ox-LDL in RPMI1640 medium supplemented with 10 % (v/v) fetal calf serum. Foam cell induction was carried out for 72 h.

### Oil red-O staining for comparison of lipid accumulation

Cultured cells were washed with phosphate-buffered saline and fixed with 10 % (v/v) formalin (Sigma–Aldrich) for 1 h at room temperature. The cells were treated with 60 % (v/v) isopropyl alcohol and incubated for 5 min at room temperature. Cells were dried and incubated with 0.21 % (w/v) oil red O staining solution (Sigma–Aldrich) for 10 min at room temperature. The cells were then washed and lipid accumulation within the cells was examined using an optical microscope (CKX-41, Olympus, Tokyo, Japan).

### Statistical analysis

Using the IBM SPSS Statistics 21.0 (IBM Corporation, Armonk, New York, USA) software, we performed age- and sex-adjusted correlations, one-way ANCOVA, one-way ANOVA and ROC analysis. In the ROC analysis, 54 individuals were all plotted for non-lipid items such as miR-33a, FBS, diastolic blood pressure and ABCA1. As a default, the statistical software chooses the optimal cut-off point across a series of cut-off points. The optimal cut-off point would be the shortest distance between the point of S_N_ = 1 and S_P_ = 1 and any points on the ROC curves$\left( \text{d}=\sqrt{\left[ {{\left( \text{1}-{{\text{S}}_{\text{N}}} \right)}^{2}}+{{\left( \text{1}-{{\text{S}}_{\text{P}}} \right)}^{2}} \right]} \right)$ [26].

## Results

### The plasma levels of miR-33a were significantly increased in athero-risk group

**Fig. 3:**
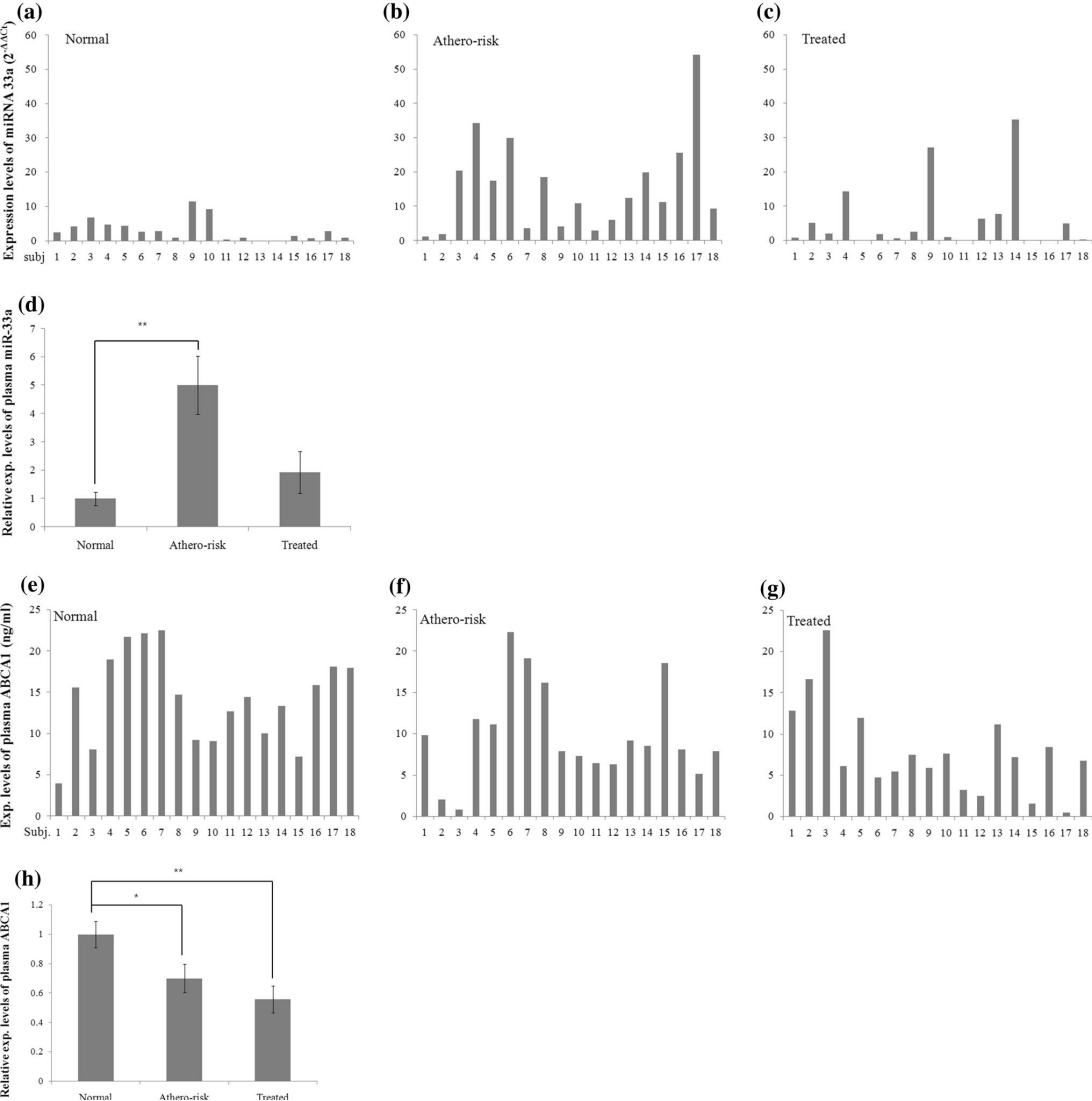
Plasma expression levels of miRNA 33a and ABCA1 in normal, athero-risk, and treated groups. The plasma expression levels of miR-33a are shown in their 2^−∆∆Ct^ values of the 18 subjects in normal, athero-risk and treated groups (**a–c**). The normalized plasma level of miR-33a of athero-risk group was 5.01-fold higher than that of normal group (*p* < 0.01) (**d**). Plasma levels of ABCA1 of 18 subjects of normal, athero-risk and treated groups are shown (**e–g**) along with their normalized ratios (**h**). Compared to the normal group, the expression levels of ABCA1 of athero-risk and treated groups decreased 0.7-fold (*p* < 0.05) and 0.55-fold (*p* < 0.01), respectively

The mean ± standard deviations of plasma levels of miR-33a (2^−∆∆Ct^) in normal, athero-risk, and treated groups were 3.15 ± 0.76, 15.77 ± 3.24, and 6.09 ± 2.34, respectively (Fig. 3a–c). Their normalized values for miR-33a were 1.0 ± 0.24, 5.01 ± 1.03, and 1.93 ± 0.74, respectively, showing that plasma miR-33a levels in the athero-risk group were significantly higher than the normal group (*p* < 0.01), while there was no significant difference between normal and treated groups (Fig. 3d).

### Plasma ABCA1 level of the treated group was lower than that of athero-risk group

Under the assumption that miR-33a suppresses the expression of ABCA1, we compared the plasma ABCA1 levels of three groups through ELISA assay. The plasma ABCA1 concentrations were 14.1863 ± 1.2853 ng/mL, 9.9295 ± 1.3694 ng/mL and 7.9358 ± 1.2952 ng/mL in normal, athero-risk and treated groups, respectively (Fig. 3e–g) showing the plasma concentrations of ABCA1 of athero-risk (*p* < 0.05) and treated (*p* < 0.01) group were lower than that of athero-risk group (Fig. 3h). Through the Fig. 3a–c, e–g, we could notice easily the antithetical expression patterns between miR-33a and ABCA1.

### Plasma miR-33a levels showed strong correlations with lipid items such as LDL and total cholesterol

In age- and sex-adjusted correlation analysis, plasma miR-33a has positive correlations with LDL and T-cholesterol (*p* < 0.01). As expected, T-cholesterol/HDL ratio has positive correlations with LDL, T-cholesterol, and TG (*p* < 0.001), but an inverse correlation with HDL (*p* < 0.001). Of note, FBS has a positive correlation with T-cholesterol (*p* < 0.05). However, we could not find an inverse correlation between the miR-33a and ABCA1 in the plasma expressions (Table 2).

**Table 2:** Age-adjusted partial correlations among lipid items, FBS, miR-33a, and ABCA1

	HDL	LDL	T-Chol	TG	TC/HDL	FBS	miR33a	ABCA1
HDL	1.000	−0.171	−0.080	−0.394**	−0.624***	0.037	0.056	0.214
LDL		1.000	0.954**	0.113	0.793***	0.266	0.363**	−0.076
T-Chol			1.000	0.340*	0.789***	0.297*	0.422**	−0.017
TG				1.000	0.514***	0.115	0.206	0.038
TC/HDL					1.000	0.118	0.265	−0.042
FBS						1.000	0.038	−0.268
miR33a							1.000	−0.016
ABCA1								1.000

*T-chol*, total cholesterol, *LDLc* low-density lipoprotein cholesterol, *HDLc* high-density lipoprotein cholesterol, *TG* triglycerides, *FBS* fasting blood sugar

*p < 0.05

**p < 0.01

***p < 0001

### miR-33a seems to inhibit the lipid efflux from cells by suppressing ABCA1 expression

Since we could not find direct causal-effects of miR-33a and ABCA1 at the human plasma level, we adopted an extrapolation of foam cell (from THP1 cell) cultures. We measured the concentration of miR-33a in the supernatant and the level of lipid accumulation caused by suppressed ABCA1. To figure out the causal-effect of the miR-33a and ABCA1, we prepared four types of foam cells through transfections of different miRNA oligonucleotides.

**Fig. 4:**
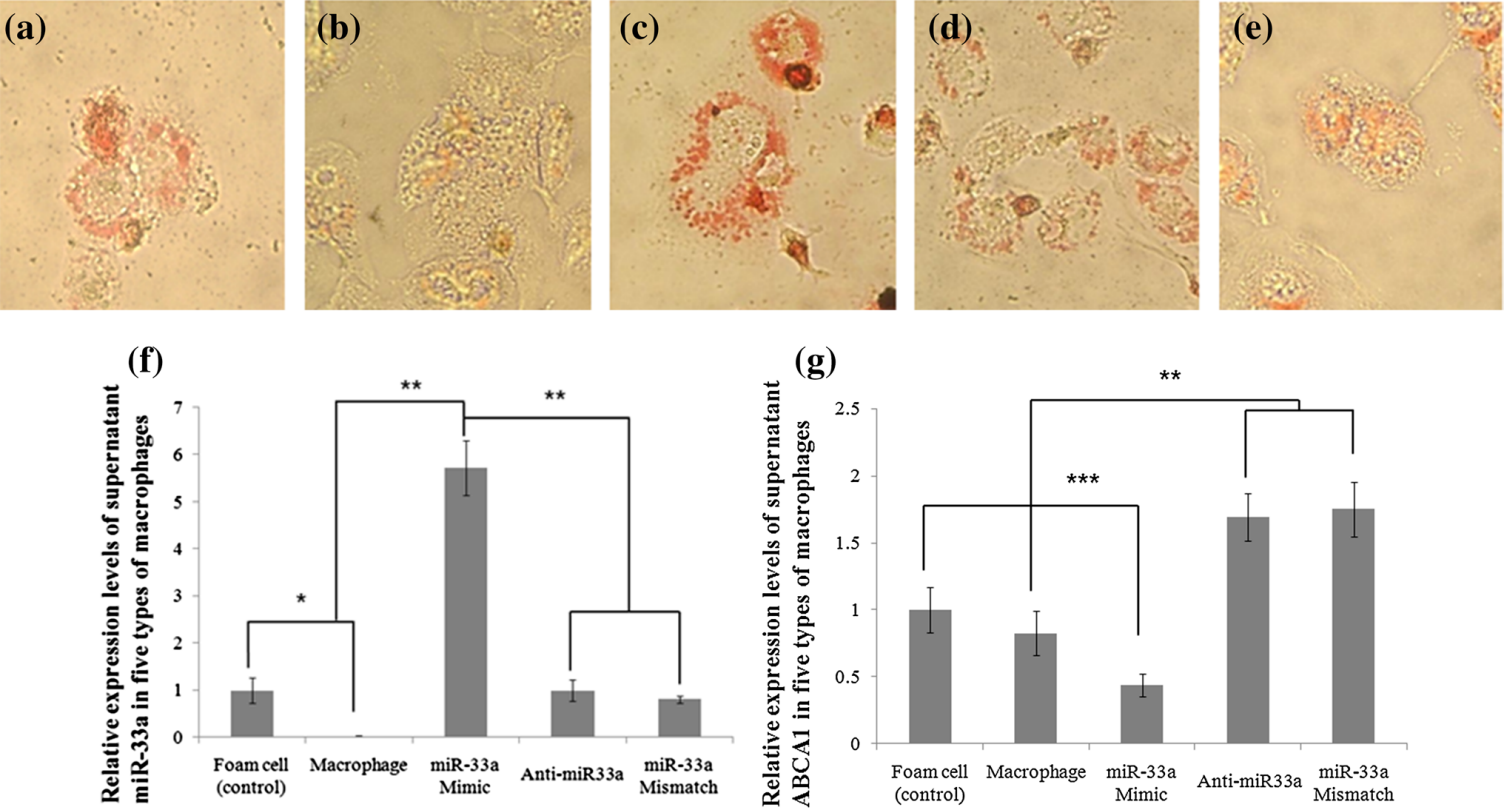
Lipid accumulation in foam cells transfected with different miRNA oligonucleotides and their secreted levels of miR-33a and ABCA1 in foam cell culture media. The five types of cells differently treated or transfected with miRNA oligonucleotides and stained with oil red O afterwards showed different levels of lipid accumulations. Moderate staining intensity in Ox-LDL-treated control foam cells (**a**), very low staining intensity in non-LDL treated macrophages (**b**), strong staining intensity in foam cells transfected with miR-33a-mimic (**c**), moderate intensities in foam cells transfected with anti-miR-33a and miR-33a-mismatched, respectively(**d–e**). Supernatant miR-33a level of foam cells that are transfected with miR-33a-mimic was 5.7-fold higher than that of control group (**f**). Supernatant ABCA1 level of cells transfected with miR-33a-mimic decreased 0.43-fold of that of control group, while those of cells transfected with anti-miR33a and miR-33a-mismatched were 1.69- and 1.75-fold higher than that in the control group, respectively (**g**)

In the comparison of lipid accumulations by oil red O staining among the four types of foam cells, the ox-LDL treated macrophages (control foam cells) showed low levels of lipid accumulation (Fig. 4a), while the non-ox-LDL treated macrophages showed a near absence of lipids (Fig. 4b). In contrast, foam cells transfected with the miR-33a-mimic showed increased staining intensity within the cells indicating greater amounts of lipid accumulation within these cells (Fig. 4c). Meanwhile, in foam cells transfected with anti-miR-33a or miR-33a-mismatched, the lipid accumulations return to the level similar to or even lower than (Fig. 4d–e) the control level (Fig. 4f–g).

Supernatant miR-33a levels were greatly increased in foam cells transfected with miR-33a-mimic. The miR-33a level was 5.86-fold higher than that of control foam cells (*p* < 0.001). Meanwhile, foam cells transfected with anti-miR-33a and miR-33a-mismatched showed 1.0- and 0.83-fold expression, respectively (Table 3; Fig. 4f).

**Table 3:** Expression levels of miR-33a and ABCA1 in the supernatant of different macrophage cultures

Macrophages and foam cells^a^	Exp levels of miR-33a^b^		ABCA1 Exp (ng/mL)	
	N	Mean ± SD	N	Mean ± SD
Foam cell (control)	3	1.00 ± 0.28***	4	4.854170 ± 0.81208**
Macrophages (non-Ox-LDL)	3	0.03 ± 0.01	4	4.010774 ± 0.81573
Foam cell with miR-33a-mimic	3	5.86 ± 0.59	4	2.117754 ± 0.41407
Foam cell with anti-miR-33a	3	1.02 ± 0.24	4	8.211216 ± 0.85186
Foam cell with miR-33a-mismatched	3	0.83 ± 0.08	4	8.504994 ± 0.98983

The expression levels were compared through one-way ANOVA

**p < 0.01

***p < 0.001

^a^Foam cells transfected with miR-33a oligonucleotides

^b^Normalized ratio

The ABCA1 levels in supernatant of cultured foam cells transfected with miR-33a-mimic decreased 0.46-fold as compared with the control foam cells (*p* < 0.001). Further, ABCA1 levels in the supernatant of foam cells transfected with anti-miR-33a and miR-33a-mismatched were increased by 1.69- and 1.75-fold, respectively, compared to the control foam cells (*p* < 0.01) (Table 3). Taken together, miR-33a might have inhibited the lipid efflux from macrophages by suppressing ABCA1 expression. The oil-red O staining seems to support the assumption that miR-33a would inhibit the cellular expression of ABCA1 (Fig. 4c–e). It seems that miR-33a suppress the expression of ABCA1 at the cellular level.

### ROC curves showed good discriminating ability of miR-33a for a candidate biomarker of atherosclerosis

**Fig. 5:**
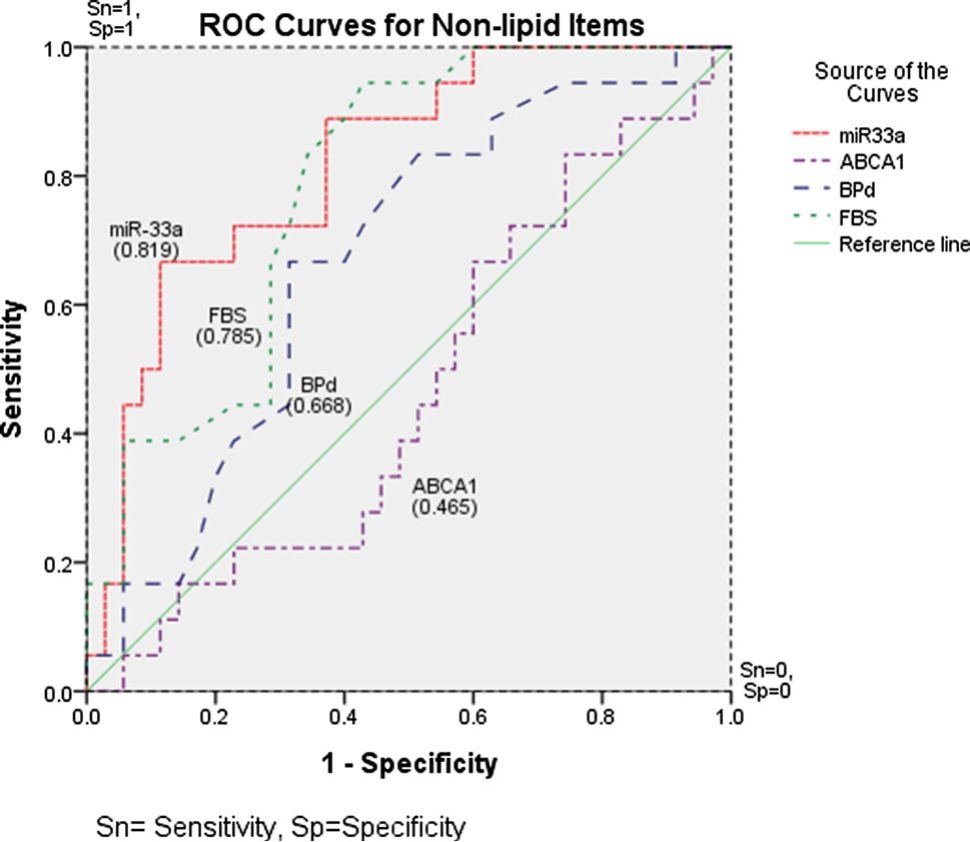
ROC curve for miR-33a as a single biomarker for the early detection of atherosclerosis. Multiple ROC curves were derived for non-lipid items. Area under curves and 95 % CIs for miR-33a, FBS, Diastolic blood pressure and ABCA1 were 0.82 (0.70–0.94), 0.79 (0.66–0.91), 0.67 (0.52–0.82) and 0.47 (0.30–0.63), respectively

AUCs (Area under curves) and 95 % confidence intervals for miR-33a, FBS, Diastolic blood pressure and ABCA1 were 0.82 (0.70–0.94), 0.79 (0.66–0.91), 0.67 (0.52–0.82) and 0.47 (0.30–0.63), respectively. Among the four non-lipid items, the plasma miR-33a showed the best discriminating ability with an AUC of 82 % as a potential biomarker for athero-risk diagnosis. In the ROC analysis for miR-33a, sensitivity, specificity, positive predictive value, negative predictive value, and odds ratio to detect atherosclerosis were 66.7, 86.1, 70.6, 83.8, and 12.4 %, respectively (Fig. 5).

## Discussion

In this study, we used a laboratory cohort data that has been developed by a diagnostic laboratory. However, the specially designed cohort could not provide us with complete information on the checkup individuals such as, type and dose of prescribed drugs and duration of treatments of the treated group.

miR-33a has been already known to suppress the cellular expression of ABCA1 in macrophages or hepatocytes [9, 10, 12, 29]. To evaluate the applicability of plasma miR-33a as a biomarker for atherosclerosis, we compared the plasma miR-33a levels among the normal, athero-risk and treated groups. Contrary to our expectation, there was no inverse correlation between miR-33a and ABCA1 at plasma level (Table 2). Moreover, the expression level of plasma ABCA1 in athero-risk group was lower than that of treated group (Fig. 3e–h). It seemed that plasma levels of ABCA1 cannot reflect the cellular expression levels of ABCA1 properly. We cast doubts on that the membrane protein ABCA1 cannot be easily transported to the plasma as well as miR-33a, which can be carried from cells to plasma more easily via microvesicles or Ago2.

Nevertheless, the expression levels of miR-33a and ABCA1 showed antithetical expressions in the culture supernatants of macrophages that are transfected with miR-33a-mimic and anti-miR-33a respectively (Table 3). The lipid accumulations shown in the oil red-o staining (Fig. 4f–g) support the antithetical expressions (Fig. 4a–e) suggesting that ABCA1 expression might be suppressed by miR-33a, at the cellular level.

Hu et al. have reported that ABCA1 expression in foam cells was suppressed by other miRNAs than miR-33a such as miR-144-3p. They found that treatment with miR-144 mimics resulted in a significant increase in lesion area of atheromatous plaque formation in apolipoprotein E^−/−^ mice. Moreover, treatment with an miR-144 mimic had an effect on the secretion of the inflammatory cytokines such as TNF-α, IL-1β and IL-6 [27]. Thus, we should investigate further how miRNAs regulates the expression of ABCA1.

In addition to miR-33a or miR-144, a few miRNAs such as miR-148a, miR-26 and miR-27a/b are known to regulate ABCA1 expression and HDL metabolism. Especially silencing miR-148a could increase the ABCA1 expression and HDL-cholesterol [28].

For quantification of miRNAs, the absolute quantification method and the construction of a standard curve may be preferable to U6 snRNA as an internal control.

FBS levels were significantly higher in athero-risk and treated groups as compared with normal group (*p* < 0.001). It is well known that aberrant expression of miR-33b is involved in the metabolism of fatty acid oxidation and insulin signaling because miR-33b targets genes such as *CPT1A, CROT, NPC, AMPK, SIRT6*, and *IRS2* [9, 14].

In conclusion, plasma levels of miR-33a in athero-risk group were fivefold higher than normal group. miR-33a seems to play an important role in inhibiting cholesterol efflux in macrophages via suppression of ABCA1 expression. Accordingly, plasma miR-33a can be a candidate biomarker for the early detection of atherosclerosis.
